# An Adaptive Hyperfine Spectrum Extraction Algorithm for Optical Sensing Based on SG Filtering and VMD

**DOI:** 10.3390/s25247590

**Published:** 2025-12-14

**Authors:** Yupeng Wu, Kai Ma, Ziyan Yun, Yueheng Zhang, Qiming Su, Xinxin Kong, Zhou Wu, Wenxi Zhang

**Affiliations:** 1 Aerospace Information Research Institute, Chinese Academy of Sciences, Beijing 100094, China; wuyupeng22@mails.ucas.ac.cn (Y.W.); makai1207@yeah.net (K.M.); yunzy@aircas.ac.cn (Z.Y.); zhangyh@aircas.ac.cn (Y.Z.); suqiming24@mails.ucas.ac.cn (Q.S.); kongxx@aircas.ac.cn (X.K.); wz@aircas.ac.cn (Z.W.); 2 School of Optoelectronics, University of Chinese Academy of Sciences, Beijing 100049, China

**Keywords:** optical sensing, spectral extraction, Variational Mode Decomposition, Savitzky-Golay filtering, parameter adaptation

## Abstract

In optical sensing, signal demodulation often degrades fine spectral data, particularly in spectroscopic measurements affected by Doppler noise, aliasing, and circuit noise. Existing algorithms often fall short in addressing these issues effectively, as they either necessitate complex parameter tuning and extensive expertise or are limited to handling simple spectral signals. To address these challenges, this study proposes an adaptive spectral extraction algorithm combining Variational Mode Decomposition (VMD) and Savitzky-Golay (SG) filtering. The algorithm optimizes parameters through an innovative adaptation strategy. By analyzing key parameters such as SG frame length, order, and VMD mode number, it leverages signal time-domain and frequency spectrum information to adaptively determine the optimal VMD modes and SG order, ensuring effective noise suppression and feature preservation. Validated through simulations and experiments, the method significantly enhances spectral signal quality by restoring absorption peaks and eliminating manual parameter adjustments. This work provides a robust solution for improving measurement accuracy and reliability in optical sensing instrumentation, particularly in applications involving complex spectral analysis.

## 1. Introduction

The molecular spectra, recognized for their broad coverage, stability, and reliability, have been extensively utilized in various fields, including material analysis, pollution monitoring, frequency standards, microchip electrophoresis, atomic structure analysis, and laser frequency stabilization [1,2,3,4,5]. Within these applications, the iodine molecule absorption spectrum, spanning from visible to near-infrared wavelengths, serves as an ideal reference for frequency-stabilized lasers. However, the fine spectral lines of iodine molecules often overlap due to Doppler broadening and other factors [3], which not only limits the precision of frequency stabilization but also poses challenges to the accuracy of sensing instruments. Moreover, environmental noise, detector noise, and circuit noise further degrade the absorption spectrum’s signal-to-noise ratio (SNR), highlighting the critical need for advanced signal processing techniques in sensing systems.

To address the demand for high-quality spectral signals in sensing applications, numerous denoising algorithms have been developed. The Least Mean Square (LMS) adaptive filtering algorithm, proposed in 2018 [6], struggles with slow convergence and a tendency to overfit, which are detrimental to real-time sensing applications. The Perfect Smoother (PS) algorithm, while capable of preserving spectral peak information, demands manual parameter estimation [7], making it less practical for automated sensing systems. Variational Mode Decomposition (VMD) offers effective spectral signal separation from noise, leveraging its high adaptability and robust theoretical foundation to reduce mode aliasing [8] and achieve more accurate spectral signal reconstruction. However, its performance remains sensitive to parameter selection, which can complicate its implementation in dynamic sensing environments. Zhou Z. from the Institute of Remote Sensing, Chinese Academy of Sciences, introduced an adaptive Savitzky-Golay (SG) filtering algorithm for satellite image processing using a quality factor [9]. In healthcare, Renfei Kuang proposed a smart photonic wristband in 2024 for pulse signal monitoring using speckle pattern analysis with a polymer optical fiber (POF), achieving a high SNR and low measurement error [10]. Despite its effectiveness in denoising and maintaining data stability and correlation, this method is not applicable to molecular absorption spectra lacking quality factor data. Other methods, such as wavelet transforms, artificial neural networks, North Crane optimization algorithms, and basis pursuit sparse denoising [11,12], have also been explored. However, these techniques are often hindered by complex parameter tuning, model intricacy, and the need for extensive expertise, making them less suitable for molecular absorption spectrum extraction in sensing applications. These limitations underscore the need for a more adaptive and robust algorithm that can enhance the precision and reliability of spectral sensing without requiring extensive manual intervention.

Composite algorithms, blending the strengths of multiple approaches, have become powerful solutions for overcoming the limitations of single algorithms in obtaining high SNR absorption spectral signals in optical sensing systems. Binary Feature Variational Mode Decomposition (F-VMD-B) has been used to eliminate interference fringes in spectral signals [13], yet its filter parameters need adjustment for different signals. Similarly, a composite noise reduction algorithm combining frequency decomposition with SG filtering can suppress large spectral peaks and extract second harmonic lines [14], but its performance is highly sensitive to SG parameters and lacks an adaptive mechanism. Fractional-order derivative (FOD) is widely used for spectral sharpening and can enhance spectral quality when combined with machine learning or swarm intelligence algorithms [1], but the resulting models are often complex and require extended training times.

To address these challenges and advance optical sensing instrumentation, this study proposes the Adaptive Variational Mode Decomposition–Savitzky–Golay Filtering (A-VMD-SG) algorithm. This algorithm dynamically determines the number of VMD decomposition modes based on the time-domain signal’s frequency spectrum and selects characteristic modes by evaluating their spectral overlap with the target. It effectively mitigates noise and adaptively determines the SG filter’s polynomial fitting order based on local inflection points within the data window, thereby enhancing noise suppression while preserving critical spectral features. By integrating advanced signal processing techniques, A-VMD-SG provides a reliable solution for improving the precision and reliability of optical sensing systems, making it a useful approach for applications requiring high-quality spectral analysis.

## 2. Methods

In this work, the hyperfine spectral line extraction algorithm is composed of three main components: signal preprocessing using the corrected power quotient, denoising with adaptive VMD, and spectral smoothing through an adaptive SG filter. The following sections provide detailed introductions to each of these components. The specific treatment effects are demonstrated through simulations and experiments in Section 3.

### 2.1. Signal Preprocessing

The spectral information is obtained using the detection light. The reference light bypasses the sample cell. By employing the correction power quotient $\;Q_{cor}$ [15], given by (1), the influence of power fluctuations on the signal can be effectively suppressed. This correction ensures that the spectral data remains accurate and reliable, even in the presence of power variations.

(1) $$Q_{cor}=\frac{P_{det}}{P_{ref}-P_{ref,DC}+\frac{P_{ref,AC}}{P_{det,AC}}P_{det,DC}}\frac{P_{ref,AC}}{P_{det,AC}}$$

where *P* represents the optical power, the subscripts “ref” and “det” denote the detection light and reference light, respectively, and the subscripts “DC” and “AC” represent the direct current and alternating current components.

After preprocessing, the residual power modulation noise in the signal is significantly suppressed, ensuring that no background noise from the modulation waveform appears during spectral sweeping. Simultaneously, the spectral details are enhanced, and the SNR is improved.

### 2.2. Denoising with Adaptive VMD

To address the boundary effect and mode aliasing problems associated with empirical mode decomposition, Dragomiretskiy and Zosso proposed VMD in 2014 [16]. Since its introduction, this algorithm and its various improved versions have been extensively applied in fields such as lidar [14] and laser absorption spectroscopy [17]. Building on this foundation, adaptive VMD leverages spectral information for parameter determination. It selects effective modes based on the overlap degree between the characteristic mode spectrum and the total spectrum, thereby eliminating low-frequency and some high-frequency noise in the spectral signal.

VMD separates the input signal *f*(*t*) into intrinsic mode functions (IMF) *u_k_* with different center frequencies, thereby achieving signal and noise separation. The VMD constraint problem can be characterized as follow [16].

(2) $$\underset{\left\{u_{k}\},\{\omega_{k}\right\}}{\min}=\left\{\sum_{k}{\left\|\partial_{t}\left[\left(\delta \left(t\right)+\frac{j}{\pi t}\right)*u_{k}\left(t\right)\right]e^{-j\omega_{k}t}\right\|}_{2}^{2}\right\},$$

(3) $$s.t.\sum_{k}u_{k}\left(t\right)=f\left(t\right),$$

where *ω_k_* represents the central frequency of the mode, and *k* is the mode number. By introducing the penalty factor α and the Lagrange multiplier λ to reconstruct the constraint equation, the convergence of the equation is improved.

(4) $$L\left(\{u_{k}\},\{\omega_{k}\},\lambda \right)=\alpha \sum_{k}{\left\|\partial_{t}\left[\left(\delta \left(t\right)+\frac{j}{\pi t}\right)*u_{k}\left(t\right)\right]e^{-j\omega_{k}t}\right\|}_{2}^{2}+{\left\|f\left(t\right)-\sum_{k}u_{k}\left(t\right)\right\|}_{2}^{2}+\langle \lambda \left(t\right),f\left(t\right)-\sum_{k}u_{k}\left(t\right)\rangle ,$$

By using the Alternate Direction Method of Multipliers (ADMM), the solution of the Lagrangian norm saddle point can be derived. The solution of the Fourier domain iterative sub-optimization is as follows.

(5) $$\begin{array}{c} {\hat{u}}_{k}^{n+1}\left(\omega \right)=\frac{\hat{f}\left(\omega \right)-\sum_{i<k}{\hat{u}}_{i}^{n+1}\left(\omega \right)-\sum_{i>k}{\hat{u}}_{i}^{n}\left(\omega \right)+{\hat{\lambda }}^{n}\left(\omega \right)/2}{1+2\alpha {\left(\omega -\omega_{k}^{n}\right)}^{2}},\\ {\hat{\omega }}_{k}^{n+1}=\frac{\int_{0}^{\infty }\omega {\left|{\hat{u}}_{i}^{n+1}\left(\omega \right)\right|}^{2}d\omega }{\int_{0}^{\infty }{\left|{\hat{u}}_{i}^{n+1}\left(\omega \right)\right|}^{2}d\omega },\\ {\hat{\lambda }}^{n+1}\left(\omega \right)={\hat{\lambda }}^{n}\left(\omega \right)+\tau \left(\hat{f}\left(\omega \right)-\sum_{k}{\hat{u}}_{i}^{n+1}\left(\omega \right)\right). \end{array}$$

where τ is the updated parameter. In this model, its initial value is 0 and it is updated iteratively at a rate of 0.01.

**Figure 1:**
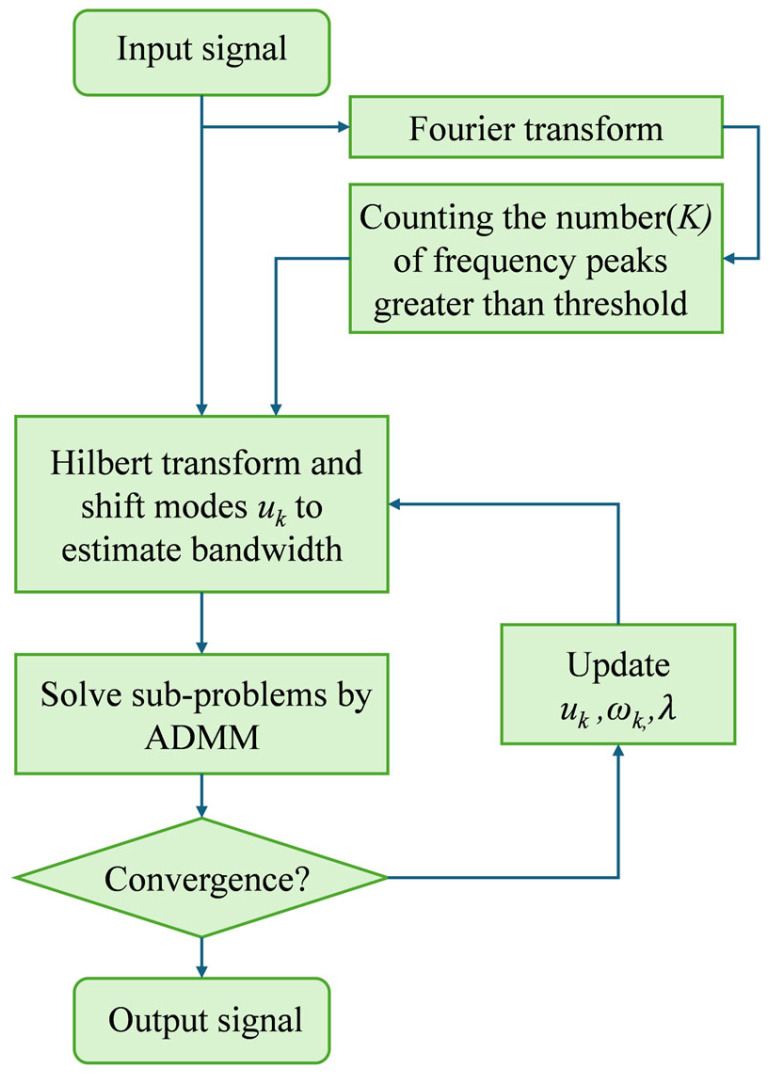
Adaptive VMD flow diagram.

Accurate determination of the number of modes *K* is crucial for restoring the contour features of absorption spectral lines. By performing a Fourier transform on the signal and observing the number of frequency peaks above a predefined threshold in the frequency domain, the number of modes in the signal can be reasonably evaluated. The flow diagram is shown in Figure 1. Furthermore, by assessing the energy distribution of these modes and eliminating those that are identified as noise, the remaining modes can be synthesized to effectively restore the spectral signal.

In the simulations and experiments conducted for this study, the parameters for VMD were configured as follows: penalty factor = 2000, absolute noise tolerance = 10^−9^, and default number of modes = 5. The adaptive VMD process is detailed in this section, with a five-frequency unequal amplitude sinusoidal noisy signal serving as an illustrative example. The mathematical representation of the simulated signal is provided below.

(6) $$S_{\mathrm{simu}}=\sin\left(2\pi t\right)+0.2\sin\left(4\pi t\right)+0.7\sin\left(14\pi t\right)+0.3\sin\left(17\pi t\right)+0.85\sin\left(20\pi t\right)+\text{noise}.$$

where *t* represents time, and “*Noise*” denotes Gaussian white noise with a mean of 0 and a variance of 0.09.

**Figure 2:**
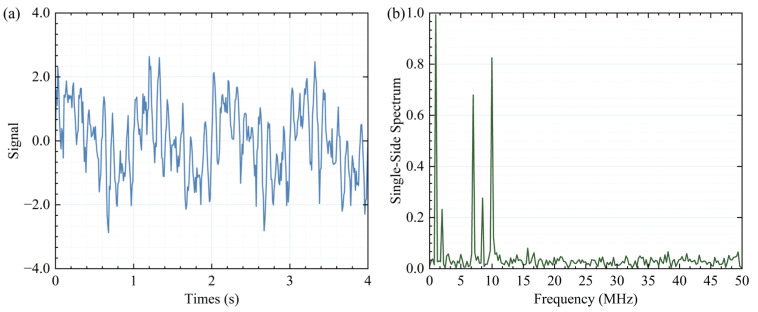
The time domain diagram (**a**) and frequency domain diagram (**b**) of simulation signal.

**Figure 3:**
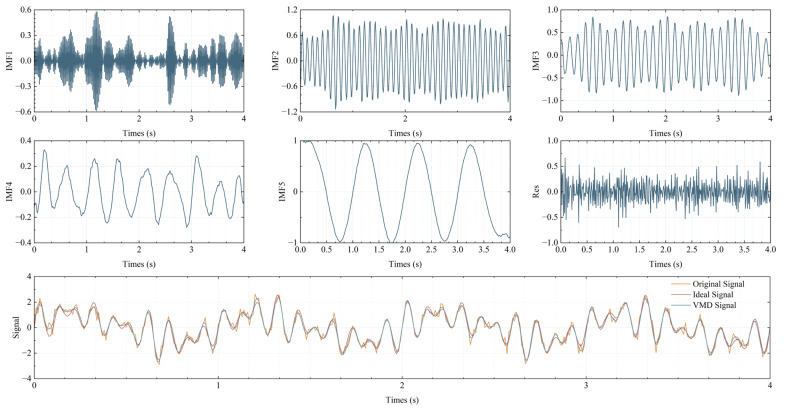
VMD signal decomposition and composition simulation.

Adhering to the widely recognized “1/10 rule,” the procedure involves incrementing the count value by 1 whenever a frequency peak with a power exceeding 1/10 of the maximum power is detected. This iterative process continues until all spectral components have been thoroughly examined, with the final count value determining the number of modes for VMD modal decomposition. The time-domain and frequency-domain representations of the signal are visually depicted in Figure 2. The frequency domain clearly reveals the presence of five distinct frequency peaks. Subsequently, the signal is decomposed into five modes, and the individual order IMFs, along with the synthesized signal, are illustrated in Figure 3. An initial estimation of the effective modes can be made based on the energy proportion of each mode.

(7) $$R_{IMF,i}=\frac{\sum_{t}E_{IMF,i}{\left(t\right)}^{2}}{\sum_{i}\sum_{t}E_{IMF,i}{\left(t\right)}^{2}}.$$

IMFs ranging from IMF2 to IMF5 display a uniform and stable energy distribution, with proportions surpassing 15%, thereby rendering them suitable for signal synthesis. Additionally, the selection of modes for synthesis can be guided by the degree of overlap between the modal spectrum and the characteristic peaks of the original spectrum. This method is employed in the simulation of spectral signals presented in Section 3. The synthesized signal effectively retains the peak and valley characteristics of the original signal, suppresses high-frequency noise, and achieves an SNR enhancement from the original 10.854 dB to 16.094 dB.

### 2.3. Smoothing with Adaptive Sg Filter

The SG filtering algorithm, proposed by Savitzky and Golay in 1964, is a digital filter based on local polynomial least squares fitting [18]. This algorithm is capable of smoothing spectra while preserving the characteristics of absorption peaks and valleys, and it has been widely applied in areas such as multi-peak spectral fitting [19], smoothing of functions obtained from empirical mode decomposition [20], and spectral smoothing of gas components [21].

The SG filter slides a fixed-length window over the signal sequence one by one, obtaining the fitted value of the central data point through local polynomial fitting. Its principle is simple and it can retain signal characteristics while suppressing noise. When using a polynomial of order p to fit 2m + 1 points, the polynomial expression is as follows:

(8) $$y=a_{0}+a_{1}x+a_{2}x^{2}+\ldots +a_{p}x^{p},$$

where $a_{j}$ represents the coefficient of the *j*-th power. By minimizing the mean square error, the solutions for each order coefficient can be obtained as follows:

(9) $$\underset{\left\{a_{j}\right\}}{\min}={\sum_{i=-m}^{m}\left(y_{k+i}-\sum_{j=0}^{p}a_{j}x_{k+i}^{j}\right)}^{2},$$

However, the filtering effect is highly dependent on the window length and the polynomial order. A small window can capture local features and is easier to achieve signal length matching. Low order fitting may overly smooth the data and lose signal characteristics, while high-order fitting may lead to overfitting. When the window length is fixed, selecting an appropriate order is particularly important. Based on the fact that a polynomial of order  p has p turning points, the appropriate polynomial order can be determined by estimating the number of turning points of the curve within the window.

(10) $$p=\text{count}\;\left(\text{number}\;\text{of}\;\text{knee}\;\text{points}\;\text{within}\;\text{the}\;\text{window}\right).$$

**Figure 4:**
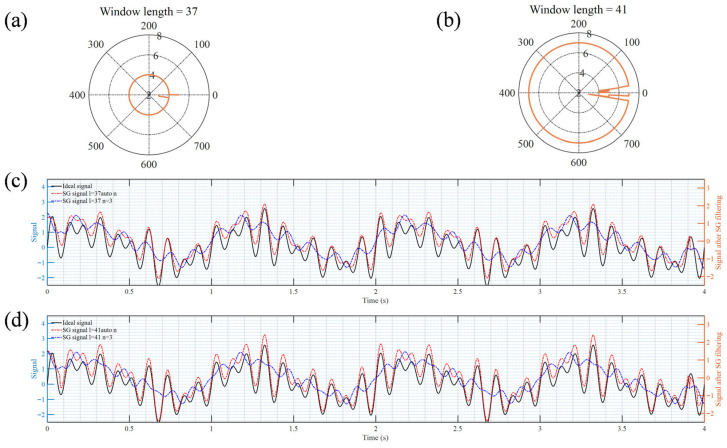
The iterative situations of polynomial order (**a**,**b**) in the SG filter window and filtering results (**c**,**d**).

In this work, the order of the SG filter is dynamically adjusted based on the number of local inflection points p within the frame length, thereby achieving higher-quality restoration of spectral peak and valley features and effectively suppressing noise in the spectral signal. The adaptive process of the SG filter is demonstrated using the sinusoidal signal group as shown in Equation (6). When the window length *l* of the SG filter is set to 37 and 41, with the default order *n* set to 3, the corresponding order iteration during each sliding window is depicted in Figure 4a and Figure 4b, respectively. As the frame length increases, the order also increases to better retain the signal characteristics. The filtering effects are illustrated in Figure 4c,d. When both the frame length and order are fixed, SG filtering exhibits issues such as excessive smoothing and feature distortion. After adopting the adaptive algorithm, the signal characteristics can be well restored. Generally, the higher the frame length, the higher the order obtained by the adaptive method.

The selection of the window length is determined by the length and characteristics of the data sequence. For shorter data sequences (e.g., tens of data points), the window length typically ranges from 5 to 11. For medium-length data sequences (e.g., hundreds of data points), the window length generally ranges from 11 to 21, approximately 1/20 to 1/10 of the data length. For longer data sequences (e.g., thousands of data points), the window length may range from 21 to 51 or larger, approximately 1/50 to 1/20 of the data length.

The criterion for identifying the inflection-point is based on the *findpeaks* function in MATLAB R2020B. After smoothing the data window, local maximum values are queried to determine the number of inflection-points. The limitations of the presented algorithm and optimization methods regarding the window length and order of the SG are explored in Section 4.

## 3. Results

To evaluate the effectiveness of the proposed method, this study conducted two types of simulations and a set of experiments: extraction of five-frequency unequal amplitude sinusoidal noisy signal, hyperfine spectral signal extraction, and extraction of iodine molecule absorption spectral signals. Initially, the effectiveness of the adaptive VMD and adaptive SG algorithms was verified using multi-frequency sinusoidal signals. Subsequently, the comparison results with traditional models were presented when processing hyperfine spectral signals. Finally, through experimental comparisons, the superiority of the A-VMD-SG algorithm was demonstrated. It should be noted that the focus of this section is to compare the accuracy, smoothing effect, and feature extraction ability of the traditional methods and the new processing method.

### 3.1. Hyperfine Spectral Signal Simulation

**Figure 5:**
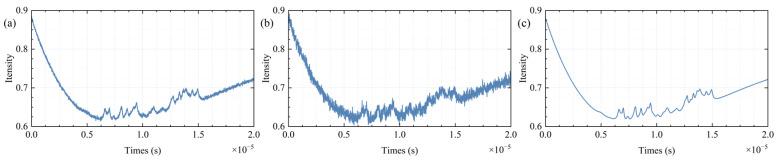
Simulated superfine absorption line with different SNR. SNR = 50 dB (**a**), SNR = 40 dB (**b**), ideal signal (**c**).

The absorption spectrum of iodine molecules is a Doppler-broadened spectral line, typically comprising several dozen lines with linewidths in the MHz range. Analysis of these lines often requires the use of non-Doppler modulation spectroscopy techniques, such as saturated absorption spectroscopy. The absorption lines can be fitted using a Lorentzian line shape, as follows:

(11) $$S\left(v\right)=\frac{\frac{2}{\pi \Delta v}}{1+{\left[\frac{2}{\Delta v}\left(v-v_{0}\right)\right]}^{2}}$$

where *v* represents the light frequency, Δv is the spectral half-width, and $v_{0}$ is the central frequency. For the simulation, 37 sets of differently parameterized Lorentzian functions were used to synthesize the simulated absorption spectral lines, with the outline shown in Figure 5. As the SNR decreases, the spectral features become increasingly submerged by Gaussian white noise. At an SNR of 40 dB, it is no longer possible to directly distinguish the spectral details.

**Figure 6:**
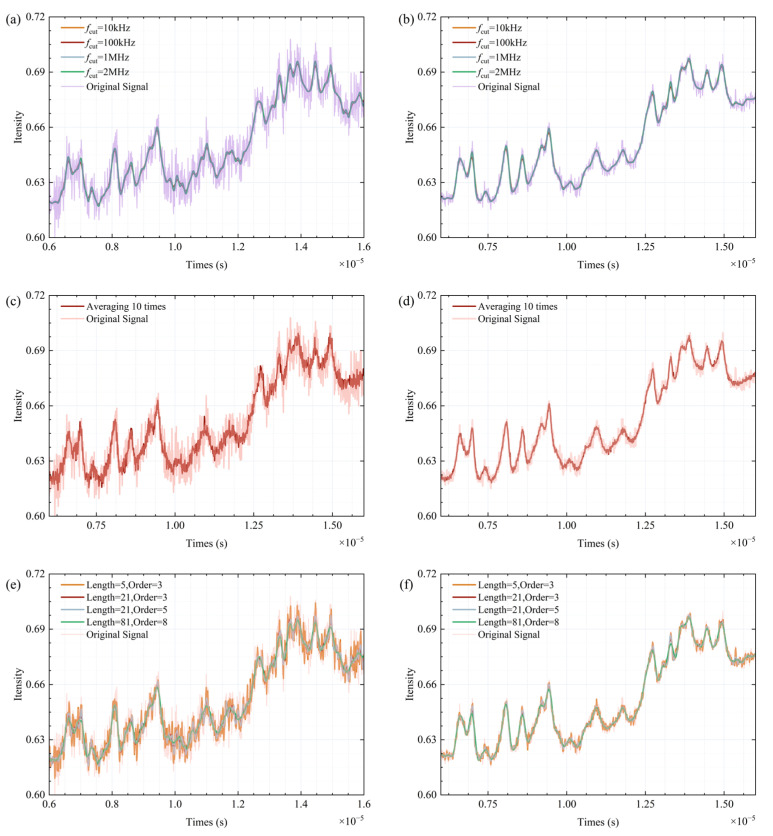
Signal of different signals after low-pass filtering (**a**,**b**), mean filtering (**c**,**d**), SG filtering (**e**,**f**). The SNR of left column and right column are 50 dB and 40 dB, respectively.

**Figure 7:**
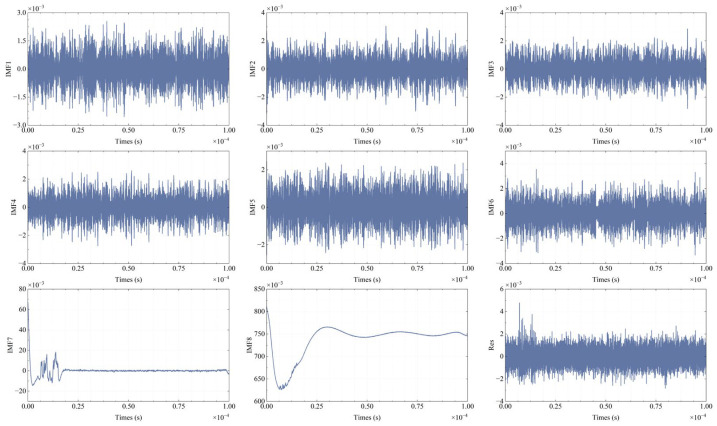
The various modes and residuals obtained after VMD (SNR = 50 dB).

**Figure 8:**
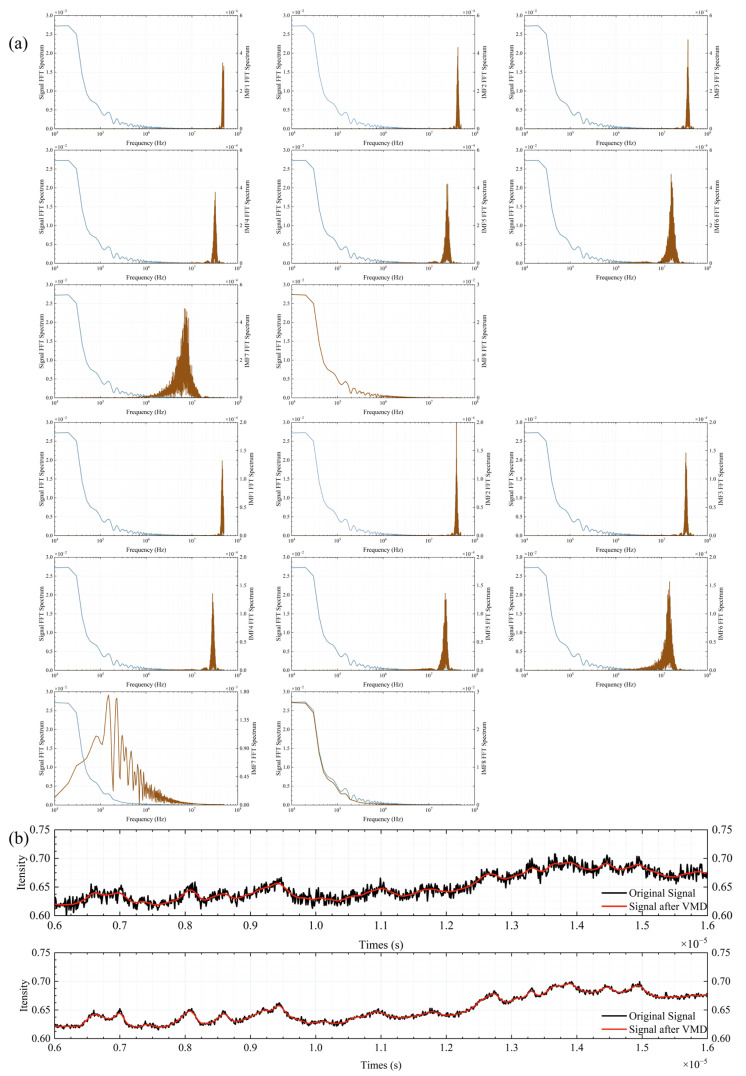
(**a**) The overlapping situation of the spectra of each IMF; (**b**) Comparison before and after VMD denoising.

**Figure 9:**
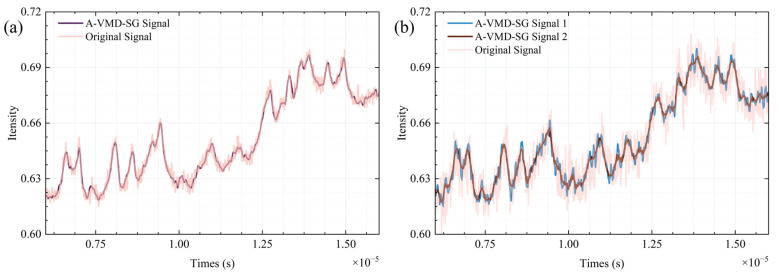
Signal of different signals after A-VMD-SG filtering. (**a**) SNR= 40 dB, (**b**) 50 dB.

The simulated spectral lines were processed through low-pass filters [22], mean filtering [23], fixed-parameter SG filtering [21], VMD synthesis denoising [17], and A-VMD-SG denoising, with the outcomes displayed in Figure 6, Figure 7, Figure 8 and Figure 9.

At high SNR, increasing the cutoff frequency of the low-pass filter brings the smoothed data closer to the ideal data. However, with a decline in SNR, the low-pass filter manifests excessive smoothing and generates false signal peaks and valleys, as depicted in Figure 6a,b. This issue remains unresolvable through mere adjustments to the cutoff frequency.

Mean filtering effectively preserves signal characteristics but requires a larger dataset for optimal performance. The improvement in the SNR is proportional to the square root of the number of cycles N in the data period. However, as the SNR decreases, residual noise becomes more pronounced, as shown in Figure 6c,d. This increase in residual noise can lead to the loss of spectral characteristics, particularly when the sampling time is extended and accumulated wavelength noise becomes significant. Additionally, when the data contains non-white noise or other types of noise, the spectral features may become blurred or even disappear during the averaging process.

SG filtering exhibits optimal performance with small-window, low-order smoothing at high SNR. At medium window lengths, the 5th-order filter surpasses the 3rd-order filter in effectiveness. Nevertheless, when the SNR decreases to 40 dB, the high-order filter with a large window continues to deliver effective smoothing while retaining signal characteristics, as shown in Figure 6e,f. Other SG filtering parameters display varying degrees of noise spikes, with the small-window, low-order filter showing almost no filtering efficacy. In low SNR conditions, while the high-order filter with a large window exhibits minor loss of signal characteristics, its performance remains markedly superior to other SG filters.

Figure 7 shows that when the SNR is 50 dB, the VMD-decomposed modal components, especially modes 7 and 8, display distinct spectral characteristics. This is supported by Figure 8a, which illustrates the spectral overlap between the IMFs and the ideal signal spectra. In Figure 8a, the blue curves represent the ideal signal spectra, while the brown curves represent the IMF spectra. The first three rows correspond to an SNR of 40 dB, and the subsequent three rows correspond to an SNR of 50 dB. At 40 dB, the IMFs from 1 to 6 predominantly capture high-frequency noise and spectral spikes, with their spectra concentrated in the high-frequency range. As the SNR increases to 50 dB, the spectral overlap of IMF7 with the ideal signal improves, indicating better noise separation. IMF8 contains most of the spectral envelope and bias. At high SNR, some spectral details shift to IMF7. Figure 8b presents the synthesized signal. The first row shows the synthesis result at 40 dB, and the second row at 50 dB. These results confirm that selecting the synthesis mode based on energy distribution allows VMD to effectively reduce noise.

In the A-VMD-SG algorithm, the threshold is designated as 1/3, with the default window length and order set at 11 and 3, respectively. Within the adaptive VMD framework, the decomposition quantity and synthesis mode are determined through spectral analysis. Subsequently, adaptive SG filtering dynamically adjusts the polynomial order in accordance with the number of inflection points within the window. The processing outcomes are exhibited in Figure 9. Simulations have demonstrated that this algorithm is capable of preserving signal characteristics across varying SNR conditions, with its noise suppression efficacy surpassing that of other effective control groups. In Figure 9, the pink curve represents the original signal, while the brown and blue curves show the A-VMD-SG processed signals. Notably, in Figure 9b, increasing the window length to 21 frames (*Signal 2*) results in a smoother curve with better preservation of spectral peak positions and intensities, highlighting the algorithm’s ability to enhance signal smoothness while retaining key spectral features.

### 3.2. Hyperfine Spectral Signal Experiment

**Figure 10:**
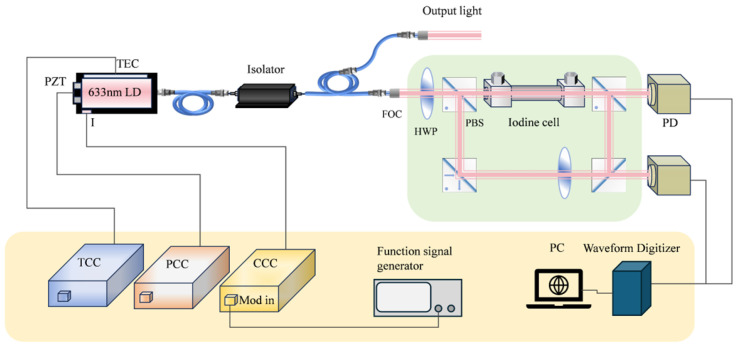
Schematic diagram of the experimental setup.

The experimental setup is detailed in Figure 10. Central to the configuration is the LD-PD INC 633-B-A81-SA-PZT laser, which serves as the primary light source. A 10 kHz modulation signal is applied to the current control circuit (CCC) to facilitate wavelength scanning. Notably, the laser exhibits a wavelength–current tuning coefficient of 1 pm/mA, allowing for fine control over its output. To ensure stability of the laser’s output, both a PZT control circuit (PCC) and a temperature control circuit (TCC) are integrated into the system. The emitted light initially traverses an isolator to prevent unwanted feedback and is subsequently divided by a 90:10 two-way coupler. Of the split light, the majority (9 mW) is directed as the output beam, while a smaller portion (1 mW) is channeled through an optical fiber collimator to serve as the experimental beam. The power of the experimental beam is meticulously adjusted using a half-wave plate (HWP) in conjunction with a polarizing beam splitter (PBS), enabling precise control over the light distribution. The beam is then split into two distinct paths, both of which pass through an iodine molecule saturation absorption system. Following this stage, the light from each path is detected by separate photodetectors, ensuring accurate measurement of the light’s properties.

**Figure 11:**
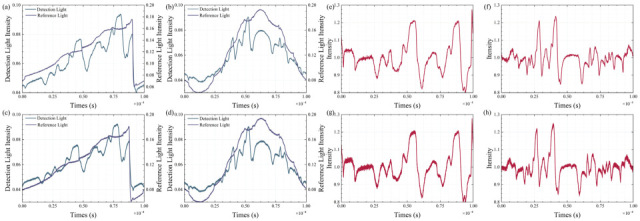
(**a**–**d**) Detection light and reference light signals. (**e**–**h**) Absorption spectral signals.

The raw signal, captured by a waveform digitizer, undergoes initial processing on the host computer. The results of this processing are presented in Figure 11. Specifically, Figure 11a–d depict four sets of preprocessed signals corresponding to the reference and detection light. Among these, Figure 11a,b were recorded at a sampling rate of 10 MSa, whereas Figure 11c,d were sampled at a higher rate of 100 MSa. After undergoing sharpening and alignment procedures, the absorption spectra derived from signals Figure 11a–d are displayed in Figure 11e,f. A striking consistency in spectral intensity, position, and width is observed across all groups, underscoring the reliability and precision of the experimental methodology. The equipment and parameters used are listed in Table 1.

In the experiment, sinusoidal and positive sawtooth waveforms were employed for spectral scanning, with a signal *V_pp_* of 1 V. The laser bias current was categorized into two groups: 89.4 mA and 103 mA. Additionally, the sampling frequencies were divided into two groups: 10 MSa and 100 MSa.

**Figure 12:**
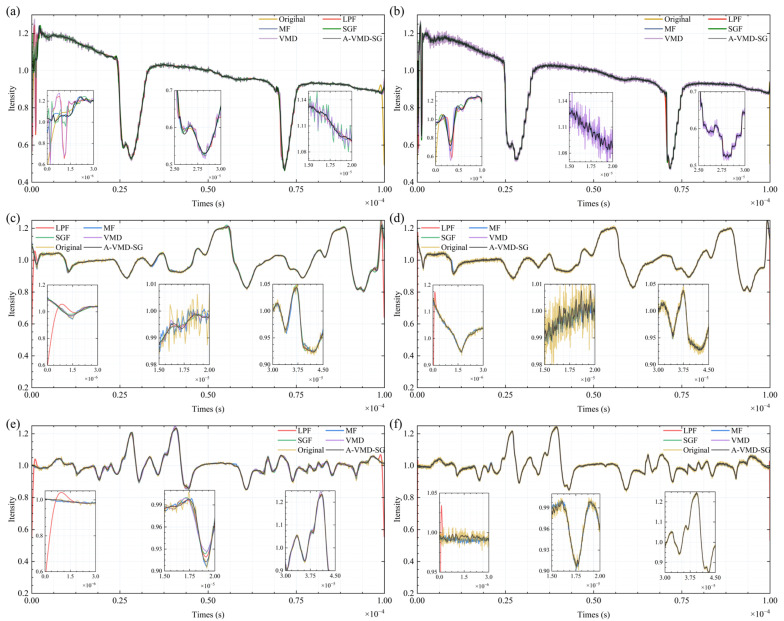
Spectral signal processing results of different algorithms. The sampling rate is 10 MSa in (**a**,**c**,**e**), and 100 MSa in (**b**,**d**,**f**). Explanation of the abbreviations in the drawings: LPF (low-pass filtering); MF (mean filtering); SGF (SG filtering); VMD (VMD synthesis denoising).

Five distinct algorithms were utilized to process the spectral signals: low-pass filtering [22], mean filtering [23], fixed-parameter SG filtering [21], VMD synthesis denoising [17], and A-VMD-SG denoising. The processing results are illustrated in Figure 12. Each of Figure 12a–f comprises one main graph and three inset graphs. In Figure 12a,b, with the laser current set at 103 mA and without the correction power ratio applied, there was evident residual power modulation noise, appearing as a negative slope sawtooth wave background. In contrast, Figure 12c–f used a laser current of 89.4 mA and incorporated a corrected power quotient to suppress power modulation noise. In these figures, the spectral features are consistent, independent of the scanning signal. The three inset graphs in each of Figure 12a–f correspond to the spectral edge, background, and absorption peak, respectively, better demonstrating the algorithm’s processing effects under different conditions. Compared to other algorithms, A-VMD-SG shows advantages in edge convergence, accurate feature extraction, and noise reduction.

In the experiment, a minimum-order finite impulse response (FIR) low-pass filter with a cutoff frequency of 100 kHz was used. The smoothed signal could generally maintain the characteristics of broad absorption peaks but exhibited excessive smoothing or divergence at sharp peaks and edges, leading to a degraded effect.

Mean filtering performs well on a short time scale and can suppress noise while preserving details. However, for longer-term data acquisition or locations with high wavelength noise, due to the cumulative wavelength errors, the processed data may lose its spectral characteristics. Additionally, since it requires a significant amount of time, occupies a large amount of storage resources, and the number of times required for the improvement of SNR increases quadratically, it is not the best solution.

The fixed-parameter SG filter was designed with a frame length of 81 points and a fitting order of 8. It could effectively smooth the data but suffered from local overfitting and deviations in the positions of absorption peaks and valleys. This method could achieve filtering improvements at high sampling rates.

For VMD synthesis denoising, while this method was capable of essentially restoring the spectral features, it exhibited a subpar performance in noise suppression. The synthesized signal displayed pronounced noise fluctuations, attributable to aliasing between the mode bandwidth and the noise bandwidth, which precluded precise separation of the signal from the noise. Although augmenting the sampling rate marginally bolstered the denoising efficacy, the resultant smoothing remained less than optimal.

In contrast, even when processing dense spectral data, such as ultra-fine spectra, A-VMD-SG demonstrated superior performance. It exhibited advantages in edge convergence, spectral detail restoration, noise suppression, and simplicity of parameter adjustment. At higher sampling rates, for the same window length, the fitting signal of A-VMD-SG was closer to the signal after 100 iterations of averaging and smoother. This finding indicates that adjusting the window length’s weight can further enhance the algorithm’s performance.

The pseudo-code of A-VMD-SG is as follows (Algorithm 1).
**Algorithm 1.** A-VMD-SG Spectrum Extraction Algorithm1 **Input:** Dual optical power intensity signal $P_{p}$, $P_{r}$.2 **Preprocessing:** Calculate the corrected power quotient.3  Fit and estimate the DC and AC component.4     $Q_{cor}=\frac{P_{det}}{P_{ref}-P_{ref,DC}+\frac{P_{ref,AC}}{P_{det,AC}}P_{det,DC}}\frac{P_{ref,AC}}{P_{det,AC}}$.5 **Adaptive parameter estimation:**6   Evaluation of the number of frequency peaks above the threshold using FFT;7  Use VMD to decompose the signal:$\underset{\left\{u_{k}\},\{\omega_{k}\right\}}{\min}=\left\{\sum_{k}{\left\|\partial_{t}\left[\left(\delta \left(t\right)+\frac{j}{\pi t}\right)*u_{k}\left(t\right)\right]e^{-j\omega_{k}t}\right\|}_{2}^{2}\right\},$
8  Evaluate the energy proportion of IMFs ($R_{IMF,i}=\frac{\sum_{t}E_{IMF,i}{\left(t\right)}^{2}}{\sum_{i}\sum_{t}E_{IMF,i}{\left(t\right)}^{2}}.$) and the spectral overlap, and select the modes to be used for synthesis;9  **for** m=1:data_length10   Count the number of knee points of the curve within the calculation window,11   Calculate the curve of the least squares fit: $\underset{\left\{a_{j}\right\}}{\min}={\sum_{i=-m}^{m}\left(y_{k+i}-\sum_{j=0}^{p}a_{j}x_{k+i}^{j}\right)}^{2},$
12   The convolution calculates the corresponding filtering value at the center point of the window.13  **end**14 **Output:** The processed spectral signal.

## 4. Discussions

### 4.1. Selection of VMD Threshold

In signal processing, the power *P* of a signal is proportional to the square of its amplitude *A* (P = A^2^). When selecting an amplitude threshold, it is essential to consider the signal’s power distribution. For a signal with a Gaussian amplitude distribution, the amplitude corresponding to 1/10 (20 dB) of the total power can be approximated as

(12) $$A_{th}=\sqrt{0.1}\approx 0.316$$

This value is close to 1/3, making 1/3 a reasonable threshold for capturing significant signal power while filtering out noise. The threshold parameter could potentially be further optimized based on specific metrics such as the SNR and Feature Preservation Index (FPI).

### 4.2. Computational Complexity

The computational and space complexities of the A-VMD-SG algorithm are analyzed to assess its feasibility for real-time sensing applications.

The computational complexity of the SG filter is dominated by the polynomial fitting and convolution operations. For a signal of length *n* and a polynomial of order *p*, the complexity of polynomial fitting is *O*(*m*·*p^2^*), where *m* is the window size. The convolution operation has a complexity of *O*(*n*). Thus, the overall complexity of the SG filter is *O*(*n*·*m*·*p^2^*). With a fixed window size *m*, this simplifies to *O*(*n*·*p^2^*). For VMD, the complexity is primarily determined by the iterative updates of the modes and center frequencies. Each iteration involves Fourier transforms with a complexity of *O*(*nlogn*) for each mode. Given *K* modes and *N* iterations, the overall complexity of VMD is *O*(*N*·*K*·*nlogn*). Combining these, the A-VMD-SG algorithm has a computational complexity of *O*(*N*⋅*K*⋅*nlogn* + *n*⋅*p^2^*). For practical applications with fixed *m* and *p*, this simplifies to *O*(*N*·*K*·*nlogn*).

The space complexity of the SG filter is determined by the storage requirements for the input signal, polynomial coefficients, and output signal. The input and output signals each require *O*(*n*) space, and the polynomial coefficients require *O*(*p^2^*) space. Thus, the overall space complexity of the SG filter is *O*(*n + p^2^*), which simplifies to *O*(*n*) for fixed *p*. For VMD, the space complexity is determined by the storage requirements for the input signal, *K* modes, center frequencies, and auxiliary variables. The input signal requires *O*(*n*) space, the *K* modes require *O*(*K*·*n*) space, the center frequencies require *O*(*K*) space, and the auxiliary variables require *O*(*K*·*n*) space. Thus, the overall space complexity of VMD is *O*(*n + K*·*n + K*), which simplifies to *O*(*K*⋅*n*) for fixed K. Combining these, the A-VMD-SG algorithm has a space complexity of *O*(*n + K*·*n + p^2^*). For practical applications with fixed *K* and *p*, this simplifies to *O*(*K*·*n*).

### 4.3. Limitations of the A-VMD-SG Algorithm

The adaptive VMD threshold is critical for determining the number of IMFs and corresponding spectral features. However, selecting an appropriate threshold is challenging. An excessively high threshold can lead to mode mixing, where distinct spectral features are merged into a single IMF, resulting in the loss of critical spectral details and reduced algorithm performance. Conversely, a threshold that is too low may produce an excessive number of IMFs, complicating subsequent analysis and decreasing computational efficiency. Therefore, identifying an optimal threshold that balances mode mixing and computational efficiency is essential for the effective application of the A-VMD-SG algorithm. Additionally, the constraint factor in VMD can be optimized by analyzing the energy proportion of noise modes in the spectral signals.

The adaptive SG filter requires careful selection of the window length based on the spectral feature length. Currently, the window length allocation is based on a rough estimation, which may not be optimal for all spectral features. A more refined approach would involve dynamically adjusting the window length according to the specific characteristics of the spectral features. However, this adaptive window length allocation increases computational time. Repeated iterations to determine the optimal window length and polynomial order can significantly slow down the algorithm’s execution, making it less suitable for real-time applications.

Regarding performance under non-stationary noise conditions, the A-VMD-SG algorithm demonstrates adaptability. The VMD component can adjust the decomposition to capture the varying frequency content of the signal, accommodating non-stationary signals to some extent. However, rapidly changing noise characteristics can still pose challenges. The SG filter can adapt to local variations in the signal, including those caused by non-stationary noise, but its effectiveness depends on the choice of window length and polynomial order. A longer window length may smooth rapid noise variations but could blur important signal features. Therefore, while the A-VMD-SG algorithm can handle non-stationary noise to a certain degree, its performance may be affected by rapidly changing noise characteristics, necessitating further research into adaptive methods for dynamically adjusting the algorithm’s parameters based on real-time analysis of the signal and noise characteristics. The spectral sharpening effect can be potentially improved by incorporating even-order spectral information into the processing method, thereby improving the signal-to-noise ratio and reducing the impact of noise on non-stationary spectra.

### 4.4. Analysis of the Spectral Extraction

**Figure 13:**
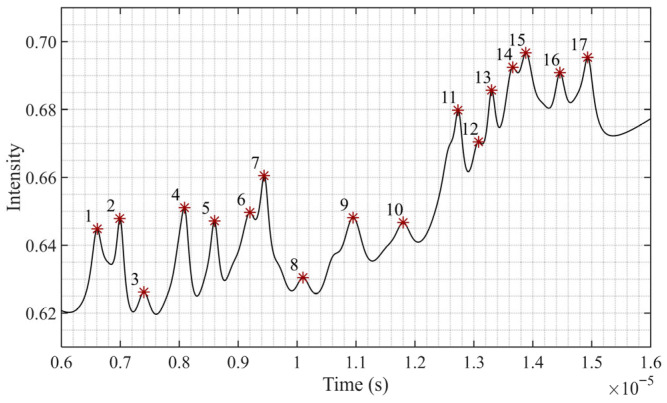
Distribution of identified peaks in the simulated spectrum. The * indicates the peak position, and the numbers indicate the peak numbers.

**Figure 14:**
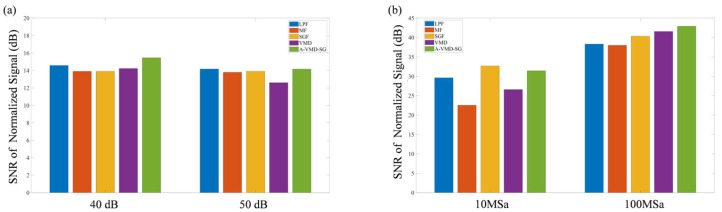
Processing effects of different algorithms under different SNRs (**a**) and sampling rates (**b**).

Due to the overlapping of fine spectral lines, the spectral simulation signal can only distinguish 17 peaks, as shown in Figure 13. Comparing the SNR values under the two sets of simulation data at 40 dB and 50 dB, as well as the two sets of spectral data with sampling rates of 10 MSa and 100 MSa, reveals that the SNR of the A-VMD-SG algorithm is superior to that of other comparison algorithms, as illustrated in Figure 14. However, due to the excessive DC component contributed by the background noise, the difference in SNR is not significant. If only the AC component is considered, the improvement in SNR will be further enhanced.

**Figure 15:**
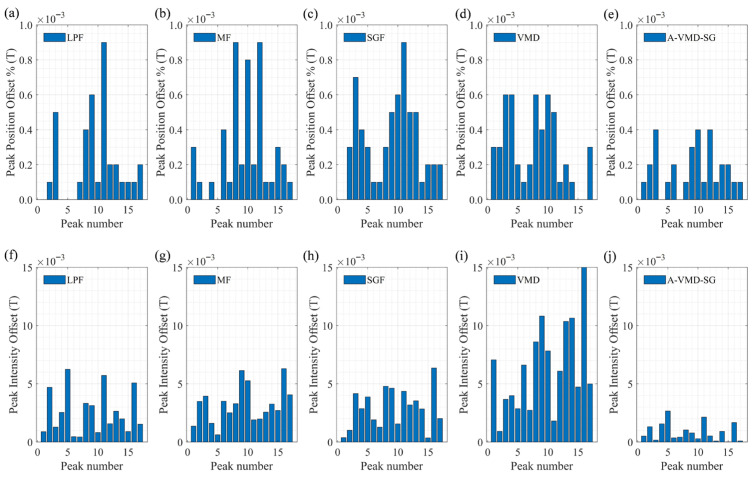
Peak position (**a**–**e**) and intensity offsets (**f**–**j**) for different algorithms at 50 dB SNR.

The Peak Position Offset and Peak Intensity Offset of different algorithms are calculated at an SNR of 50 dB. The A-VMD-SG algorithm demonstrates significantly better performance than other algorithms, with a peak position offset of less than four parts per million of the sampling length and a peak intensity offset of less than three parts per thousand, as detailed in Figure 15.

**Figure 16:**
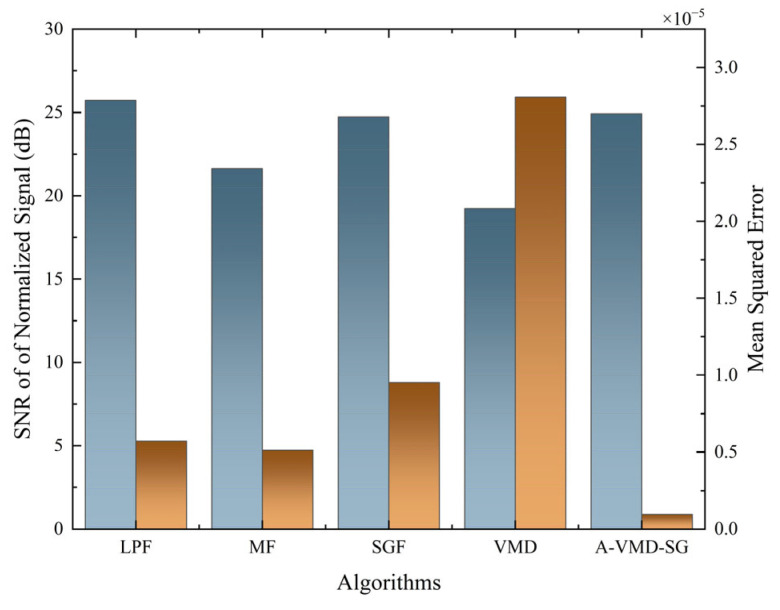
SNR and reconstruction errors for different algorithms at 50 dB SNR.

To evaluate the signal reconstruction effect, the mean square error (MSE) is used for assessment. The data is shown in Figure 16. Even though the SNR of other algorithms is close to that of A-VMD-SG, their reconstruction errors are much worse, indicating that A-VMD-SG can also perform well in the spectral transition region for smoothing.

## 5. Conclusions

This study presents an adaptive spectral extraction algorithm combining Variational Mode Decomposition and Savitzky-Golay filtering to address the challenges of spectral noise in optical sensing. Spectral noise significantly impacts the intensity of absorption peaks and valleys, leading to distortions in spectral line shapes and potential misjudgments in peak and valley positions. This results in decreased frequency stabilization accuracy, incorrect component analysis, and unreliable frequency standards.

The main contributions of this work include:

1. Adaptive Algorithm Development: An adaptive algorithm based on VMD and SG filtering is proposed for high-quality spectral signal extraction. The algorithm autonomously acquires model parameters through local signal processing, eliminating the need for additional model training.
2. Power Fluctuation Correction: A reference light-based power fluctuation correction method is introduced, utilizing a correction quotient to suppress the impact of power fluctuations on measurements, thereby enhancing instrumentation accuracy.
3. Parameter Optimization: The algorithm evaluates VMD parameters based on spectral information and SG filter parameters based on local window information. The threshold parameter simplifies adjustments and demonstrates insensitivity to signal length, enhancing the algorithm’s adaptability in practical instrumentation scenarios.
4. Enhanced Spectral Signal Features: By eliminating power noise and applying smoothing techniques, the algorithm yields high-quality spectral signals that improve measurement reliability and precision.
5. Algorithm Validation: The effectiveness and superiority of the A-VMD-SG algorithm are validated through comparisons with different optimization algorithms. Both simulations and experiments confirm its capability to restore high-quality spectral signals, providing a robust solution for spectral signal processing in instrumentation applications.

In summary, this study provides a robust solution for improving measurement accuracy and reliability in optical sensing instrumentation, particularly in applications involving complex spectral analysis.

## Figures and Tables

**Table 1:** Table Experimental parameters settings.

Equipment	Parameter	Brand
Laser	-	LDPD-INC
Constant current source	89.4 mA/103 mA	Thorlabs LDC202C
Temperature control device	10 kΩ	Thorlabs TED200C
Detector	-	Thorlabs PDA36A2
Iodine cell	Φ20 × 100 mm	CellI2-801
Signal generator	-	Moku: pro
Waveform Digitizer	10 MSa/100 MSa	AlazarTech ATS9462

## Data Availability

The data related to this study can be requested from the corresponding authors.
